# Fear of hypoglycaemia in parents of young children with type 1 diabetes: a systematic review

**DOI:** 10.1186/1471-2431-10-50

**Published:** 2010-07-15

**Authors:** Katharine Barnard, Sian Thomas, Pamela Royle, Kathryn Noyes, Norman Waugh

**Affiliations:** 1 National Institute for Health Research Health Technology Assessment Programme, University of Southampton, Southampton, SO16 7NS, UK; 2 Department of Public Health, University of Aberdeen, Foresterhill, Aberdeen, AB25 2ZD, UK; 3 Royal Hospital for Sick Children, Sciennes Road, Edinburgh, EH9 1LF, UK

## Abstract

**Background:**

Many children with type 1 diabetes have poor glycaemic control. Since the Diabetes Control and Complications Trial (DCCT) showed that tighter control reduces complication rates, there has been more emphasis on intensified insulin therapy. We know that patients and families are afraid of hypoglycaemia. We hypothesised that fear of hypoglycaemia might take precedence over concern about long-term complications, and that behaviour to avoid hypoglycaemia might be at the cost of poorer control, and aimed to evaluate the effectiveness of any interventions designed to prevent that. The objective of this review was to systematically review studies concerning the extent and consequences of fear of hypoglycaemia in parents of children under 12 years of age with type 1 diabetes, and interventions to reduce it.

**Methods:**

Data Sources: MEDLINE, EMBASE, PsycINFO, The Cochrane Library, Web of Science, meeting abstracts of EASD, ADA and Diabetes UK, Current Controlled Trials, ClinicalTrials.gov, UK CRN, scrutiny of bibliographies of retrieved papers and contact with experts in the field.

Inclusions: Relevant studies of any design of parents of children under 12 years of age with Type 1 diabetes were included. The key outcomes were the extent and impact of fear, hypoglycaemia avoidance behaviour in parents due to parental fear of hypoglycaemia in their children, the effect on diabetes control, and the impact of interventions to reduce this fear and hypoglycaemia avoidance behaviour.

**Results:**

Eight articles from six studies met the inclusion criteria. All were cross sectional studies and most were of good quality. Parental fear of hypoglycaemia, anxiety and depression were reported to be common. There was a paucity of evidence on behaviour to avoid hypoglycaemia, but there were some suggestions that higher than desirable blood glucose levels might be permitted in order to avoid hypoglycaemia. No studies reporting interventions to reduce parental fear of hypoglycaemia were found.

**Conclusions:**

The evidence base was limited. Parents of children with Type 1 diabetes reported considerable parental fear of hypoglycaemia, affecting both parental health and quality of life. There is some suggestion that hypoglycaemia avoidance behaviours by parents might adversely affect glycaemic control. Trials of interventions to reduce parental anxiety and hypoglycaemia avoidance behaviour are needed. We suggest that there should be a trial of structured education for parents of young children with Type 1 diabetes.

## Background

Type 1 diabetes mellitus is one of the most common childhood diseases. It is characterised by hyperglycaemia, caused by an absolute insulin deficiency [1]. Type 1 diabetes is usually diagnosed in childhood and adolescence and is the leading form of diabetes in young white people, especially those of northern European ancestry [2]. The incidence has been increasing in children younger than 15 years, trebling in Scotland over the last 30 years. The biggest relative increase has been in those under 5 years old. It is predicted that in European children under the age of 5 years, the incidence rate will double between 2005 and 2020, with prevalent cases younger than 15 years rising by 70% [2].

The long-term complications of diabetes include two forms of vascular disease. Micro-vascular disease causes retinopathy, nephropathy and neuropathy (which increases the risk of foot ulcers and sexual dysfunction). Most people will develop microvascular complications after 15-20 years of poorly controlled diabetes [3].

Macrovascular disease causes heart disease and stroke. Heart disease is the main cause of death in type 1 diabetes in developed countries [4]. There is also evidence that chronic hyperglycaemia (particularly in young boys) could be related to poorer neuro-cognitive outcomes. Evidence from the Diabetes Control and Complications Trial [5] and its long-term follow-up study, the Epidemiology of Diabetes Interventions and Complications (EDIC) study [6], have shown that a period of poor control can cause lasting damage - the "metabolic memory" effect-even if control later improves. It is therefore important to aim at good control from diagnosis.

Scottish audit data showed that only 10% of children were meeting the NICE guideline target for blood glucose control [7]. Similar data from England and Wales showed that 19% of children in participating centres achieved that target. This data shows that 81% of both 0-5 year olds and 6-11 year olds, fail to achieve a target HbA1c of less than 7.5% [8]. Furthermore, 28% of children under 12 years old have very poor control, i.e. above 9.5% HbA1c.

Following evidence from the DCCT [5] that indicated improved control by intensive insulin treatment reduced complication rates, there has been a drive towards more intensive insulin regimens. These involve not just more injections, but regular self-measurment of blood glucose, self-adjustment on insulin dose, and attention to diet and other lifestyle factors. One consequence of the intensified control in DCCT was a marked increased in the frequency of hypoglycaemia [9]. In very young children, there is concern that severe hypoglycaemia can cause some cognitive impairment [10]. Hypoglycaemia is one of the most important barriers to good glycaemic control.

For young children, most care comes from parents who administer or oversee treatment [11]. Up to the age of 8 years, parents have sole responsibility for diabetes management tasks (or securing suitably qualified or experienced childcare), whilst between ages 8-11 children begin to take over some of those tasks. By adolescence, a negotation occurs between parents and adolescents about the transfer of responsibility for diabetes with the main burden of care being lifted off parents [12]. Hence this review focuses on the under 12s only.

Diabetes management often has negative consequences on parents' own well-being. New routines must be introduced and maintained, new knowledge learned and coping strategies developed to adapt to life with a child with Type 1 diabetes. Diabetes-related management practices, time demands, finances, social support, independence, stigma and concern for the future must all be balanced with achieving optimal glycaemic control and quality of life (QoL) for their child [13].

Children and their families 'learn quickly that hypoglycaemic episodes are physically aversive, potentially dangerous and a source of possible social embarrassment'[14]. As such, they 'acknowledge significant anxiety about the occurrence of hypoglycaemic episodes and maintain marginally elevated blood glucose levels and engage in premature treatment of apparent hypoglycaemia'[15]. The associated risks of long term complications may be overshadowed by short term goals to avoid hypoglycaemia. This "hypoglycaemia avoidance behaviour" in the parents may lead to poorer glycaemic control, in their children, increasing the risk of long-term complications.

There has been no previous systematic review of this area. The objective of this study is to systematically review studies evaluating the fear of hypoglycaemia in parents of children under 12 years of age with type 1 diabetes, assess the effect on hypoglycaemia avoidance behaviour and on glycaemic control, and identify interventions which are effective in reducing fear of hypoglycaemia and hypoglycaemia avoidance behaviour.

## Methods

The review was carried out systematically, following the general principles recommended by the Centre for Reviews and Dissemination (CRD 2009) [16].

### Inclusion criteria

#### Study design

Study designs of any type were eligible for inclusion.

#### Types of participants

Parents (or other primary carers) of children under 12 years with type 1 diabetes. Children on any insulin regimen were included.

### Outcomes

• The extent of parental fear of hypoglycaemia

• The effect of parental hypoglycaemia avoidance behaviour on child's glycaemic control as reflected in HbA1c or frequency of hypoglycaemic episodes or admissions for metabolic derangements

• The effect of parental fear of hypoglycaemia on parent's quality of life, anxiety, and depression,

• The impact of any intervention aimed at reducing parental fear of hypoglycaemia and hypoglycaemia avoidance behaviour.

### Search strategy

The search strategy comprised the following main elements:

• Searching of electronic databases: The Cochrane Library (Issue 1, 2010); MEDLINE (1950-March 2010); Embase (1980-March 2010); Science Citation Index Expanded (1970-March 2010); Social Sciences Citation Index (1970-March 2010); Conference Proceedings Citation Index-Science (1990-present); PsycINFO (1967-March 2010).

• Meeting abstracts of European for the Study of Diabetes (EASD); American Diabetes Association (ADA); Diabetes UK.

• Current Controlled Trials; ClinicalTrials.gov; UK Clinical Research Network

• Contact with experts in the field

• Scrutiny of bibliographies of retrieved papers

No language or date restrictions were applied to the search. Further details of the full search strategy are reported in Appendix I

### Study selection

**Figure 1:**
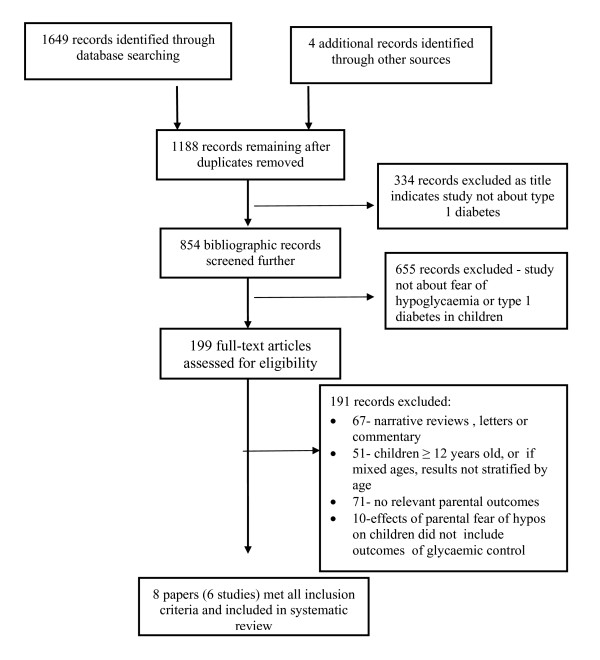
**Flow Diagram of Selection Process**.

Titles and abstracts were examined for inclusion by two reviewers. Full copies of papers which appeared to fulfil the inclusion criteria (or where there was doubt) were obtained and were independently selected by two reviewers for inclusion in either phase of the review. Disagreements were resolved by discussion (see Figure 1 for inclusion/exclusions).

### Quality Assessment

The quality of each study was assessed using tools appropriate to the study design: based on the 'Crombie criteria' for assessment of cross-sectional studies [17] adapted by Petticrew and colleagues[18]. Quality was assessed independently by two reviewers. Disagreements were resolved through discussion.

### Data extraction

Data were extracted independently by one reviewer using a standardised data extraction table and checked for accuracy by a second reviewer. Disagreements were resolved through discussion and with reference to the original article.

### Data analysis

A meta-analysis was not possible due to the lack of data and the differences in populations and outcome measures. Studies were, therefore combined in a narrative synthesis. Possible reasons for conflicting results were also reported narratively.

Differences by treatment (multiple daily injections versus insulin pump therapy versus conventional regimens) were to be explored in subgroup analysis but treatment regimen was not reported in most of the studies.

## Results

Of 1649 abstracts originally screened, 199 met the initial inclusion criteria for retrieval of full papers for further investigation. Following extensive screening, only eight papers from six studies met the full inclusion criteria, with no intervention studies applicable for inclusion. All articles reported cross-sectional studies with one cross sectional/correlational study. Quality Assurance data is presented in Appendix II. Results of data extraction are presented in tables Appendix III. The studies in this section will be referred to by the surname of the first author and year of the study.

Additional file 1 contains data extraction results in Tables 1, 2 and 3.

### Demographic Data

The number of parent/caregiver participants taking part ranged from 24 to 114 (mean = 79). The number of child participants was reported in four studies Patton 2008 [19], Clark 1998 [20], Mueller-Godeffroy 2009 [21] and Marrero 1997 [22], and was 81, 46, 38 and 32 respectively, with an earlier paper, (Patton 2007 [23]), reporting results in a subset of 24 on CSII. Female parents/caregivers made up 100% of participants in the Jaser 2009 study [24]; dropping to 82-84% in Patton 2007 [23] and 60% in Patton 2008 [19]. One study, Mitchell 2009 [25], reporting a subset of the participants in the Monaghan 2009 study [26] included only male parent/caregivers as participants.

The ages of participating children ranged across studies from 2 to 11 years (means 4.45 +/- 1.5 to 5.7 +/- 1.8 years). The duration of the children's diabetes was less than 3.5 years in all studies, ranging from one month to five years.

### Insulin regimens

The insulin regimens reported included insulin pump therapy, basal bolus/multiple daily injections and conventional 2-3 injections a day. HbA1c was reported in five studies, Patton 2008 [19], Clarke 1998 [20], Mueller-Godeffroy 2009 [21], Jaser 2009 [24], and Monaghan 2009 [26], and ranged from 6.0% to 11% (mean 8.19%).

### Fear of hypoglycaemia

The commonest instrument used was the Hypoglycaemia Fear Survey (HFS) with the parent version (HFS parent) being used in all studies.

Mothers of young children with Type 1 diabetes in the Patton 2008 study reported greater fear of hypoglycaemia than fathers of young children (p = .006) and higher scores on the behavioural subscale, (p = .001), but there were no statistically significant differences between mothers and fathers on the worry subscale[19]. Mitchell 2009 [25] reported low levels of hypoglycaemic fear in fathers (mean = 16.7, range 0-44). Greater paternal paediatric parenting stress however, in the Mitchell study, was correlated with fathers' psychological resources including lower self efficacy about diabetes management (r = -.46, p =< .01), more fear of hypoglycaemia (r = .43, p =< .01), more state anxiety (r = .67, p =< .001) and less hope (r = -.60, p =< .001).

Half of participants in the Patton 2007 study reported the child experiencing an episode of hypoglycaemia 3-5 times per week [23]. However, the Patton 2008 study reported that severity of hypoglycaemia was more important in causing fear than frequency, especially in parents whose child had experienced a hypoglycaemic seizure. They also found that almost a third of children (32%) had experienced at least one hypoglycaemia seizure during their lifetime [19]. Marrero 1997 [22] reported that parents of children who had experienced a hypoglycaemic seizure within the past year had significantly greater overall fear of hypoglycaemia (both behaviour and worry scales) than those whose children had not experienced a seizure. Furthermore, children who had experienced a seizure with loss of consciousness had a significantly higher percentage of self monitoring of blood glucose (SMBG) values above the desired target range than young children with no history of seizures (p = 0.03). Clark 1998 [20] supports this finding in that mothers whose children had a history of passing out had significantly higher HFS scores than mothers whose children had never lost consciousness (79.6 +/- 13.9 versus 70.2 +/- 14.7, p = .040). Patton 2007 [23] reported similar findings, although not statistically significant, with parents of young children who had seizures worrying more than parents of children who had not (50.7 +/- 12.6 versus 41.7 +/- 9.6 respectively). In the Marrero 1997 study there was no significant correlation between the parental HFS total score and parental DQoL general worry about their child having diabetes (r = 0.34, p =< 0.06) [22].

Mothers' level of fear (as assessed by the HFS) did not relate to the number of hypoglycaemic episodes over the previous twelve months in Clarke 1998 [20]. However, Monaghan 2009 reported that mothers' level of fear was related to their degree of distress over hypoglycaemic episodes that occurred when their child was asleep (r = .372, p = .005) or in social situations (r = .279, p = .03); but was not related to maternal confidence in their ability to treat hypoglycaemia or to their confidence at being able to recognise hypos [26].

Patton 2007 reported that the most common fears reported by parents relating to hypoglycaemia were feeling the child will have a low blood glucose while asleep (63% of participants), and the child having a low blood glucose when away from a parent (46%) [23]. Additional results from this study suggest that parents of children with higher average blood glucose levels reported greater fear of hypoglycaemia (p = 0.05), with a trend between parents' worry score and children's daily blood glucose control (p = 0.06).

### Anxiety and depression

Increased maternal depression and anxiety were associated with greater fear of hypoglycaemia in Jaser 2009 [24]. This study also reported, however, that maternal symptoms of anxiety and depression were not related to child's metabolic control. Although surprisingly, in most children HbA1c was below the treatment goals recommended for their age group (average HbA1c 6.86% SD 0.86).

Patton 2008 reported that parental anxiety includes "not being there" if the child needs them whilst they are asleep alongside concerns that their child will 'have a hypo' during the night [19].

### Socio-economic influences

Patton 2007 found that negative associations were reported between families' socioeconomic status and total/worry scales on the HFS (p < 0.001 and p =< 0.05 respectively). Also, family income and education was related to mother's coping resources (i.e. resources currently available to individuals for managing stress) [23]. Further evidence of socioeconomic influence were found by Jaser 2009, who reported that lower income levels and finding it more upsetting to cope with diabetes related stress, accounted for higher symptoms of anxiety and stress in mothers. Monaghan 2009 reported that Caucasian parents and those with higher education reported greater fear associated with hypoglycaemia. Fewer years of education and lower income were reported to produce a greater state anxiety in Patton 2007 and Monaghan 20 [23,26].

Stallwood reported that parents with children who maintained HbA1c within a target range had higher incomes and knowledge levels than those with children with HbA1c levels outside target range (p =/< 0.05 and p =/< 0.01 respectively) [27].

### Hypoglycaemic avoidance behaviour

Patton 2007 reported that parents of children with higher than average blood glucose levels were reported to engage in frequent use of behaviours aimed at preventing hypoglycaemia as assessed by the HFS PYC behaviour score (p = 0.04).

The higher scores in mothers on the behavioural subscale of the HFS indicated greater use of maladaptive coping behaviours to avoid hypoglycaemia (such as 'have my child eat large snacks at bedtime' and 'allow my child's blood glucose to be a little high to be on the safe side' items on HFS) in Patton 2008 [19].

Common strategies used by parents to prevent hypoglycaemia in Patton 2007 were carrying fast-acting sugar (100%), checking blood glucose often when attending a long event (75%), avoiding being away from their child when his/her blood glucose might go low (67%), feeding the child at the first sign of hypoglycaemia (63%) [23].

Monaghan 2009 reported that parents often engage in nocturnal blood glucose monitoring, and those who reported 'often/always' were more likely to have a child on a basal-bolus regimen and their child having significantly longer illness duration (p =< 0.05) [26].

## Discussion

We found that the evidence base on fear of hypoglycaemia amongst parents, and its effect on their quality of life, is quite limited. However, fear of hypoglycaemia was common, especially amongst mothers. Hypoglycaemic episodes are quite frequent, but the fear of hypos is related more to the severity of hypoglycaemia than the frequency, especially if the child has had a hypoglycaemic convulsion. Parental anxiety and depression are common. However, hypoglycaemic avoidance behaviour was rarely reported, perhaps because it is a very sensitive topic. We heard anecdotal evidence from personal communication with healthcare professionals at Southampton and Leeds Paediatric Diabetes Centres that some parents have a tendency to run blood glucose levels 'slightly' higher than recommended to avoid acute episodes of hypoglycaemia, resulting in higher HbA1c. Parents, especially mothers, the main carers, may seek to avoid hypoglycaemia as indicated on the behaviour subscale of the HFS-P [19,28].

Parental fears include fear of hypoglycaemia and associated seizures; anxiety associated with frequent blood glucose monitoring; fear of 'not being there'; general anxiety associated with the burden of caring for a child with Type 1 diabetes and fear that others, such as babysitters, teachers and others will be unable to provide appropriate care for their child [29]. Nocturnal hypoglycaemia in particular poses particular stressors to mothers, irrespective of the number of hypoglycaemic episodes reported. Monaghan [26] reports that frequency of nocturnal blood glucose monitoring was positively associated with parent reported anxiety and parenting stress (p =< 0.05).

One problem if blood glucose is allowed to run chronically high is that the signs and symptoms of acute hypoglycaemia may present at higher blood glucose levels i.e. above 4 mmol rather than 3.9 and below, when the patient is actually not hypoglycaemic. This can happen when blood glucose has dropped from a higher reading or when blood glucose readings are very variable.

The risk of severe hypoglycaemia can be minimised by appropriate self-monitoring of blood glucose, regular review of insulin regimen, review of the quantity and timing of carbohydrate intake, and the use of CSII [12].

A Norwegian study was excluded from this review because it covered an age range up to 15, with about 40% of children outside our age range of interest, and did not give results split by age band [28]. Parents of 115 children participated, completing the Hypo Fear Survey Parent version. Mothers scored significantly higher than fathers on both worry and behaviour subscales (p =< 0.001) suggesting that mothers may engage in hypoglycaemia avoidance behaviours. Parents of children receiving insulin injections scored higher than parents of children using CSII on the behaviour subscale (P =< 0.001) but for all parents, severe hypoglycaemia in a child is one of the most important causes of fear. The level of fear was not correlated with the number of hypoglycaemic episodes, but was related to their severity, especially in mothers of children who have experienced a hypoglycaemic seizure. The authors conclude that future interventions should target both parental fear and appropriate ways to prevent hypoglycaemia in children with Type 1 diabetes [28].

Overall, the methodological quality of the cross sectional studies (as shown in Table 1 in Appendix III) was good. All studies were recruited appropriately, from paediatric diabetes clinics. In four studies it seemed that participants in the study were a representative sample of the clinic population, whereas in the other two studies it was unclear. One study (Mueller-Godeffroy 2009 [21]) met all seven quality indicators, three studies met six criteria, and two met four criteria. The most common methodological limitation was the lack of a power calculation to justify whether the sample size used in the study was adequate. Only two studies reported doing such a calculation. The other limitation in some studies was the response rate. We defined a good response rate as 70% or more, in line with good practice recommendations [30]. Two studies did not report the response rate, and of the remainder, three were good and one, Jaser 2009 [24] was low (40%). This could be a possible source of bias if non-responders differ in some way with respect to the variable being measured.

The main limitation of the current review was the very limited evidence base. Furthermore, the complex and multifaceted issues affecting parental fear of hypoglycaemia still require further exploration. There were few papers that specifically addressed the issue of fear of hypoglycaemia separately from wider parental anxiety associated with the diagnosis and treatment of a child with Type 1 diabetes. This is a highly sensitive area and it could be that well designed, robust qualitative research is required to tease out parental attitudes is a more sensitive way than could be achieved using quantitative methods.

We need to know if there are interventions which could alleviate the burden of fear associated with hypoglycaemia. Could a structured education course for parents help? In the UK, parents of children aged up to 12 years with Type 1 diabetes currently do not receive any structured education on how best to manage their child's biomedical and psychosocial needs. There is no evidence of whether a structured education programme for parents of young children would be helpful. Better understanding of the disease might overcome any tendency of parents to allow poor glycaemic control because of fear of hypoglycaemia.

Structured education programmes have been used for older patients. DAFNE (Dose Adjustment for Normal Eating) is a 5-day out-patient course for adults with Type 1 diabetes, teaching the skills of carbohydrate counting and insulin dose adjustment. An RCT of DAFNE reported improvements in glycaemic control, satisfaction with treatment, and quality of life compared to those receiving standard care [31].

A structured education programme, KICk-OFF(Kids in Control of Food (KICk OFF), for 11 to 16 year olds with Type 1 diabetes based on the DAFNE principles, is currently the subject of an RCT. The pilot study of the course found significant improvements in quality of life, satisfaction with diabetes treatment and self-efficacy on child and parent reports, 6-months after completing the course. Glycaemic control in those with poor control at baseline also improved at 6-months post-course [32].

Parents from lower socioeconomic backgrounds report finding it harder to cope [23,24], thus interventions may needed to be tailored to take account of these differences.

## Conclusion

Parents of a child with Type 1 diabetes report a high level of anxiety and fear associated with managing the condition. Further research is needed to determine whether fear of hypoglycaemia leads to poorer glycaemic control because of hypoglycaemia avoidance behaviour, and whether structured education courses for parents would be effective in reducing worry and improving control.

## Abbreviations

ADA: American Diabetes Association; CRD: Centre for Reviews and Dissemination; DAFNE: Dose Adjustment for Normal Eating; DCCT: Diabetes Control and Complications Trial; DQoL: Diabetes Quality of Life; EASD: European Association for the Study of Diabetes; EDIC: Epidemiology of Diabetes Interventions and Complications; HFS: Hypoglycaemia Fear Survey; HFS PYC: Hypoglycaemia Fear Survey Parents of Young Children; KiCk OFF: Kids in Control of Food; QoL: Quality of Life; SD: Standard Deviation; SMBG: Self Monitoring of Blood Glucose

## Competing interests

The authors declare that they have no competing interests.

## Authors' contributions

PR designed the search criteria, conducted the electronic searches and initial screening. NW, ST and KB screened abstracts. ST and KB extracted data, KB wrote the first draft and subsequent revisions. KN provided expert advice and reviewed the article. PR, NW and ST contributed to writing of paper and reviewing. All authors read and approved the final manuscript.

## Appendix I

The MEDLINE search strategy (below) was adapted as appropriate for other databases

1. exp Diabetes Mellitus, Type 1/

2. type 1 diabet*.tw.

3. (child* adj2 diabet*).tw.

4. 1 or 3 or 2

5. exp Parents/

6. (maternal or mother* or paternal or father* or famil* or parent or parents or carer*).tw.

7. 6 or 5

8. exp Hypoglycemia/

9. hypoglyc*.tw.

10. 8 or 9

11. 4 and 7 and 10

## Appendix II

### Quality Assessment

Cross sectional studies

1. Was the research design appropriate?

2. Was the recruitment strategy appropriate?

3. What was the response rate?

4. Is the sample representative?

5. Were objective and reliable measurements used?

6. Was there a power calculation or justification of numbers?

7. Was the statistical analysis appropriate?

8. Was there evidence of bias in the study?

## Pre-publication history

The pre-publication history for this paper can be accessed here:

http://www.biomedcentral.com/1471-2431/10/50/prepub

## Supplementary Material

Additional file 1 **Data Extraction Tables**. summary of data extraction from selected papers. Click here for file
