# Supplementary material for: Fear of hypoglycaemia in parents of young children with type 1 diabetes: a systematic review
Source: BMC Pediatr. 2010 Jul 15;10:50. doi: 10.1186/1471-2431-10-50 (PMC2912881; doi:10.1186/1471-2431-10-50)
Supplement: Additional file 1 — Data Extraction Tables. summary of data extraction from selected papers. [file 1471-2431-10-50-S1.DOC]

**TABLE 1 - QUALITY ASSESSMENT OF CROSS SECTIONAL** STUDIES

| **Author and year** | **Appropriate Research Design?** | **Appropriate Recruitment Strategy?** | **Response Rate?** | **Is Sample Representative? (All clinic populations)** | **Objective and Reliable Measures?** | **Power Calculation/ Justification of Numbers?** | **Appropriate Statistical Analysis?** | **Evidence of Bias?** | **Quality Indicators Met** |
| --- | --- | --- | --- | --- | --- | --- | --- | --- | --- |
| Clark 1998[20] | Yes | Yes | Not reported | Unclear | Yes | No | Yes | Authors state representative of clinical population | 4/7 |
| Jaser 20009[24] | Yes | Yes | 40% | Unclear | Yes | No | Yes | No evidence of clinical difference between populations | 4/7 |
| Marrero 1997[22] | Yes | Yes | Not reported | Yes (of clinic population) | Yes | Yes | Yes | Convenience sample | 6/7 |
| Monaghan 2009[26]; Mitchell  2009[25] | Yes | Yes | 71% | Yes (Mitchell subset of Monaghan study) | Yes | No | Yes | Mitchell reports subset of Monaghan study results –not clear how subset recruited | 6/7 |
| Mueller-Godeffroy  2008[21] | Yes | Yes | Approx. 76% | Yes (of clinic population) | Yes | Yes | Yes | Convenience Sample | 7/7 |
| Patton 2007 and 2008[19,23] | Yes | Yes | 85% (73% in 2008 study) | Yes (of clinic population) | Yes | No | Yes | One clinic in large urban children’s hospital; 2007 reports subset of results from 2008 study | 6/7 |

**APPENDIX IV**

**DATA EXTRACTION TABLES**

**Table 2 - Study Characteristics**

| **First Author** | **Study Design** | **Number of Participants** | | **% Female**  **Parent/caregiver** | **Age of Children (Mean)** | **Duration of Child’s Diabetes (Mean)** | **Insulin Regimen** |
| --- | --- | --- | --- | --- | --- | --- | --- |
|  |  | **Parents/**  **Caregivers** | **Children** |  |  |  |  |
| Clark 1998[20] | Cross sectional | 46 | 46 | 100% | 8.1 +/-2.2 | 3.4 yrs +/-2.0 | 2 subcutaneous injections daily |
| Jaser 2009[24] | Cross sectional | 67 | Unclear | 100% | 4.8 years +/- 1.5 | 1.4 years +/- 1.3 | 68% on CSII |
| Marrero 1997[22] | Cross sectional | 31 | 32 | 92% | 8.6 years (SD 3.4) | Not stratified by age | Not known |
| Monaghan 2009[26]  Mitchell  2009[25] | Cross sectional  These results were published separately | 43 | 43 | Fathers only | 2-6 yrs (mean 4.5, SD 1.1) | 6 months to 3.7 years, Mean 1.3 years SD, 1.0, | 70% conventional two to three insulin injections per day; 25% basal bolus regimens and 5% insulin pump |
|  |  | 71 | Unclear | 97% |  | 1.47 years (SD 1.09) | Conventional insulin regimen of 2 to 3 injections daily 79%: basal-bolus insulin 17% on multiple daily injections; 4% on insulin pump; |
| Mueller Godeffroy 2008[21] | Cross sectional | 29 | 25 (aged 8-11) | Unclear | 4-7 and 8-11 | Unclear for age groups | Unclear for age groups |
| Patton 2007 and 2008[19,23] | Cross sectional  (These results were published separately) | 24 | 24 | 83% | 5.7 years +/-1.8 | 3.1 years +/-1.3 | Continuous subcutaneous insulin infusion (CSII) |
|  |  | 81 mothers/64 fathers | 81 | 60% | 5.6 years (SD 1.6) | 3.4 years SD 1.5 | conventional insulin regimen of 2-3 injections a day (n=57); insulin pump therapy (n=24) |

**Table 3 - Outcome Measures**

| **First Author and Date** | **HbA1c** | **Frequency of Hypoglycaemia** | **Parent Outcomes** | **Other Outcomes** | **Assessment Methods** | **Results** |
| --- | --- | --- | --- | --- | --- | --- |
| Clark 1998[20] | 10.8+/-2.0% | 32.6% had passed out with hypoglycaemia in the past | Hypoglycaemia Fear, Worry and Behaviour | Demographic variables;  HbA1c | - Modified Hypoglycaemia Fear, Worry and Behaviour Scale – Parent (HFS-P) | - Mothers whose children had passed out during hypos had significantly higher HFS-P scores than mothers whose children had never lost consciousness (79.6 +/-13.9 vs 70.2+/- 14.7, p=0.040) |
| Jaser 2009[24] | 6.86 (SD 0.86) | Not reported | Current symptoms of depression and anxiety; fear of hypoglycaemia; coping and demographic variables | Child’s clinical data; demographic variables | - Center for Epidemiological Studies – Depression - State-Trait Anxiety Inventory - Issues with Coping with Insulin Dependent Diabetes Mellitus – Parent Scale - Hypoglycaemia Fear Survey - HbA1c | - Maternal symptoms of depression and anxiety - CES-D mean 10.86(SD6.98); State Anxiety mean 35.09 (SD10.88); ICC Coping Upset mean 47.03 (SD12.71); Fear of Hypoglycaemia mean 14.96 (SD9.59) - Psychological symptoms – Mothers meeting cut-off score for clinically significant symptoms of anxiety (STAI-S >44) n=14 (20.9%); and depression (CES-D score 16 or more) n=16 (24.2%). - There were no significant group differences on any key variables between child gender, age, clinical variables (insulin via pump or injections) or psychosocial variables of mother (fear of hypoglycaemia, coping, anxiety and depression). - Higher levels of maternal anxiety symptoms were related to finding diabetes more upsetting and harder to cope with and greater fear of hypoglycaemia. Maternal symptoms of anxiety and depression were not related to child’s metabolic control. |
| Marrero 1997[22] | Not known | Not reported | Fear of hypoglycaemia; parental behaviour dependent on seizure with loss of consciousness (SLC) | SMBG; HbA1c (not stratified by age range) | - Hypo Fear Survey - Parental DQoL | - Non significant correlation between Parental HFS total score and parental DQoL general worry about their children having diabetes (r=.34, p=<.06). - Parents of children who had experienced a hypoglycaemic seizure within the past year had a significantly greater overall fear of hypoglycaemia as reflected in both HFS parental behaviour and worry scales - Parents of children with history of seizure did not test their children’s blood glucose more frequently than parents of children who had not had a seizure - Children who had experienced SLC had a significantly higher % of SMBG values above the desired target range than young children with no history of SLC events (p=.038) |
| Monghan 2009[26]; Mitchell 2009[25] | 9.5% (SD 0.9) | Not reported | Parenting stress | Demographic data, HbA1c, and medical outcomes | - Paediatric Inventory for Parents - Hypo Fear Survey - Eyberg Child Behaviour Inventory - Self Efficacy for Diabetes Scale | - Fathers’ perceived difficulty of paediatric stress was mild (M=82, scale range 42-210). - Fathers’ psychological resources – reported relatively high self efficacy (M=92, scale range 22-110) and hope (M=72.1m scale range 12-76). - Fathers reported low levels of fear of child experiencing hypoglycaemia (M=16.7, scale range 0-44) and state anxiety (M=35.4, scale range 20-80). - Greater paternal paediatric parenting stress was correlated with father’s psychological resources, including lower self efficacy about diabetes management, more fear of child experiencing hypoglycaemia, more state anxiety and less hope. |
|  | Average HbA1c = 7.49% (SD=1.06) reported as “well controlled”.  (These results published separately) | Seizures/blackouts due to hypoglycaemia (% with at least one event) 13% | Frequency of nocturnal blood glucose measurement (NBGM); Fear of hypoglycaemia; state anxiety; paediatric parenting stress |  | Parental outcomes recorded through structured interview; Hypoglycemia Fear Survey; State subscale of the State-Trait Anxiety Inventory (STAI-S); the Paediatric Inventory for Parents (PIP); | - Maternal education, child ethnicity and family income were significantly correlated with HFS Worry and STAI-S scores (p<0.10). Caucasian parents and those with higher education reported greater fear of hypoglycaemia and parents with fewer years of education and lower income reported greater state anxiety. - Mothers’ level of fear (as assessed by the HFS) did not relate to the number of hypoglycaemic episodes over the previous twelve months however. Mothers level of fear was related to mothers degree of distress over hypoglycaemic episodes that occurred when their child was asleep (r=.372, p=.005) or in social situations (r=.279, p=.03). - General frequency of NBGM: rarely/never (n=23); sometimes (n=25); often/always (n=23). Frequency of NBGM was positively associated with parent reported anxiety and parenting stress (p<0.05). |
| Mueller Godeffroy 2008[21] | Age 4-7: Pre: 7.4+/-1.38% (range 3.7 to 10.3); post 7.3+/-1.08% (range 4.7 to 9.4);  Age 8-11 years: Pre 7.6 +/- 0.78% (range 5.8 to 9.2); post 7.4 +/- 0.85% (range 5.7 to 9.4) | Not reported as stratified by age range | Family burden; fear of hypoglycaemia | HbA1c | - KINDL-R and KINDL-DM QoL measures - Paediatric Inventory for Parents - Hypo Fear Survey Parental Version - Behavioural Paediatrics Feeding Assessment Scale | - Baseline data not reported but at 6 month follow up parents of younger children (aged 4 to 7) reported significantly less frequent parenting stress (difference -4.72 (95% CI -22.20 to -7.24, p=0.000); less difficulty with parenting stress in the total Scores of PIP (difference -1.83; 95% CI -19.76 to -3.90; p=0.005); and in all PIP subscales although the decreases were not statistically significant for communication frequency or difficulty. Significant decrease was reported in hypoglycaemia related worries (HFS-P Worry scale) of -7.69 (95% CI -11.49 to -3.90, p=0.0001) but there was no significant decrease in the HFS-P Behaviour scale; there was less frequency of feeding behaviour problems (BPFAS-F) - Parents of school-aged children (age 8 to 11 years) also reported a significant decrease in fear of hypoglycaemia: Worry: mean difference -7.69 (95% CI -11.49 to -3.90, p=0.0001); however the difference in the Behaviour subscale was not statistically significant (mean difference 1.42, 95% CI: -1.46 to 4.30, p=0.319). |
| Patton 2007 and 2008[19,23] | Mean 206 mg/dL daily +/-14 | History of hypoglycaemic seizure Yes 25%; No 85%.  Frequency of hypoglycaemia (<60 mg/dL) Once per day 4%; 1 or 2 per week 33%; 3-5 per week 50%; Once per month 9%; once every few months 4%. | Fear of hypoglycaemia | HbA1c | - Modified Hypoglycemic Fear Survey Parents of young children (HFS-PYC) | - Fear of Hypoglycaemia: Parental mean total score (HFS-PYC: 81 +/- 14.1; Parental mean Worry and Behaviour subscale scores 44.3+/1 11.1; - Negative associations were reported between families’ level of socioeconomic status and parental Total and Worry scores (p<0.01 and p<0.05 respectively). There was a trend (though not statistically significant) in parents’ HFS-PYC Worry scores suggesting parents of young children who had seizures may worry more about hypoglycaemia than parents whose child had not had a seizure (50.7+/- 12.6 and 41.7+/-9.6 respectively). - Results suggested that parents of children with higher average blood glucose levels engaged in frequent use of behaviours aimed at preventing hypoglycaemia as assessed by HFS-PYC Behaviour score (p=0.04). - Common strategies used by parents to prevent hypoglycaemia were: carrying fast-acting sugar (100%); checking blood glucose often when attending a long event (75%); avoiding being away from child when his/her blood glucose might go low (67%); feeding the child at the first signs of hypoglycaemia (63%). - Most common fears reported by parents relating to hypoglycaemia were: feeling the child will have low blood glucose while asleep (63%) and the child having a low blood glucose when away from parent (46%). - Associations between parents fear of hypoglycaemia and child’s average daily blood glucose levels: Results suggested that parents of children with higher average blood glucose levels reported greater fear of hypoglycaemia (p=0.05). There was also a trend (though not statistically significant) between parents’ Worry Score and children’s daily blood glucose control (p=0.06). No correlation found between parents’ HFS-PYC Behaviour scores and children’s average daily blood glucose levels (apart from during study period). - HFS-PYC scores correlated negatively with % of blood glucose tests within target range (p<0.05 for total, worry and behaviour scales), and positively with % of tests above the target range (p<0.05 for total, worry and behaviour scales). No significant correlation between parents’ HFS-PYC scores and % of blood glucose tests below the target range. |
|  | Daily 207 mg/dl (SD 49)  (These results published separately) | Mean 5.5 (SD1.9) during study period | Parental fear of hypoglycaemia |  | - Modified Hypoglycemic Fear Survey Parents of young children (HFS-PYC) | - There was a positive correlation reported between mothers’ scores on worry subscale of HFS-P-YC and reporting of frequency of hypoglycaemic events (p<0.05). - No significant correlations were found for mothers HFS-P-YC total and behaviour scores and children’s frequency of hypoglycaemic events; mothers’ HFS-P-YC scores and children’s mean daily blood glucose levels, number of hypoglycaemic events within study period and HbA1c levels. - Higher total HFS-P=YC scores were reported for mothers of children who had a positive seizure history compared with mothers of children who had never had a seizure (p=0.05). Mothers of children who had a hypoglycaemic seizure reported higher scores on the behaviour subscale than mothers of children who had never had a seizure, although this difference did not reach statistical significance. - Comparison of Mothers’ and Fathers’ HFS-P-YC Scores: Mothers of young children with type 1 diabetes reported greater fear of hypoglycaemia than fathers of young children (p=0.006) and higher scores on behaviour subscale (p=001). However no statistically significant differences were reported between mothers and fathers on the worry subscale. - The higher scores in mothers on the behavioural subscale of the HFS indicated greater use of maladaptive coping behaviours (such as ‘have my child eat large snacks at bedtime’ and ‘allow my child’s blood glucose to be a little high to be on the safe side’ items on HFS) to avoid hypoglycaemia |
